# A Case of Acute Myeloid Leukemia (FAB M2) with Inversion 16 Who Presented with Pelvic Myeloid Sarcoma

**DOI:** 10.1155/2014/246169

**Published:** 2014-12-22

**Authors:** Mustafa Çakan, Ahmet Koç, Kıvılcım Cerit, Süheyla Bozkurt, Rabia Ergelen, Irmak Vural

**Affiliations:** ^1^Department of Pediatric Hematology and Oncology, Faculty of Medicine, Marmara University, Mimar Sinan Caddesi No. 41, Pendik, 34899 Istanbul, Turkey; ^2^Department of Pediatric Surgery, Faculty of Medicine, Marmara University, Mimar Sinan Caddesi No. 41, Pendik, 34899 Istanbul, Turkey; ^3^Department of Pathology, Faculty of Medicine, Marmara University, Mimar Sinan Caddesi No. 41, Pendik, 34899 Istanbul, Turkey; ^4^Department of Radiology, Faculty of Medicine, Marmara University, Mimar Sinan Caddesi No. 41, Pendik, 34899 Istanbul, Turkey; ^5^Department of Pediatrics, Faculty of Medicine, Marmara University, Mimar Sinan Caddesi No. 41, Pendik, 34899 Istanbul, Turkey

## Abstract

Acute leukemias are the most common childhood cancer in all age groups. Acute myeloid leukemias (AML) constitute about 15–20% of acute leukemias. Fatigability, pallor, fever, and bleeding are the most common presenting symptoms of AML. Hepatosplenomegaly and lymphadenopathy are commonly encountered during physical examination. In rare instances eruptions due to skin involvement and localized tumor masses (myeloid sarcoma) may be found. Myeloid sarcoma is especially seen in AML-M2 subtype. By cytogenetic analysis, in AML-M2 subtype t(8;21) is often seen and it is more probable to find inversion 16 in AML-M4Eos subtype. Herein, we present a 15-year-old girl whose initial symptom was abdominal pain for three days and her pathological sign was a large abdominal mass which was verified by imaging studies and diagnosed as myeloid sarcoma by biopsy. On bone marrow examination, she had diagnosis of AML-M2 and by cytogenetic analysis inversion 16 was positive. She was treated with AML-BFM 2004 protocol and she is being followed up in remission on her ninth month of the maintenance therapy.

## 1. Introduction

Acute myeloid leukemia (AML) comprises 15–20% of all childhood acute leukemias. In most of the patients, fever, pallor, weight loss, and mucosal bleeding are seen. In more than half of the cases, liver, spleen, and lymph nodes are palpable [1, 2].

Myeloid sarcoma (chloroma and granulocytic sarcoma) is defined as a tumoral mass which is formed by immature myeloid cells in the extramedullary area. It is seen in less than 5% of all AML cases. The most common locations are head and neck region, skin, gingival region, and intracranial and paravertebral areas [3–6].

In FAB classification, AML is classified according to myeloblasts' morphology into 10 subtypes [7]. In 2008, WHO classification was developed and AML was reclassified according to accompanying cytogenetic abnormalities [8]. AML-M2 comprises 10–15% of all AML cases. Myeloid sarcoma may accompany AML-M2 more commonly than other subgroups. By cytogenetic analysis t(8; 21) is often found in this subgroup. It is more probable to see inversion 16 [inv(16)] in AML-M4Eos [1, 2, 9].

## 2. Case Report

**Figure 1:**
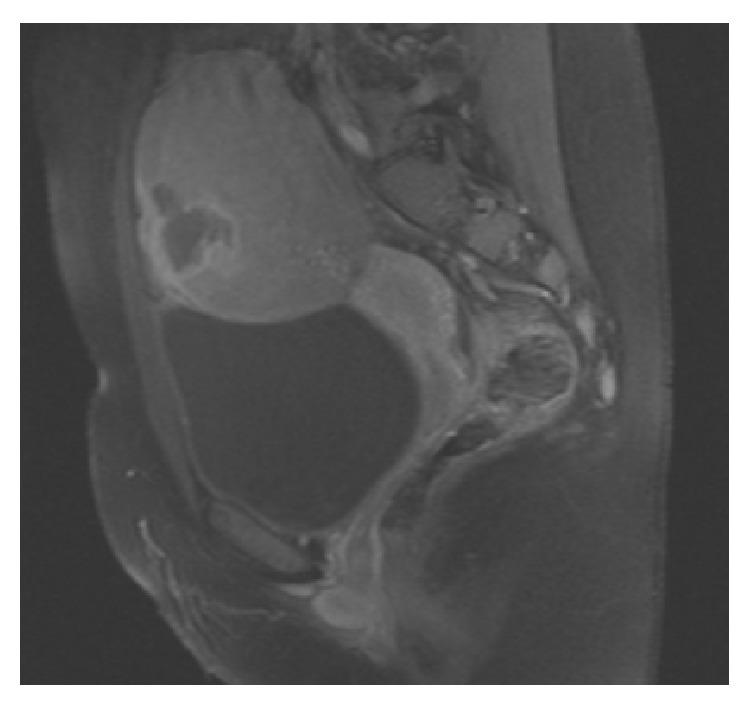
Sagittal T2-contrast-enhanced, fat saturated MR image of the mass.

**Figure 2:**
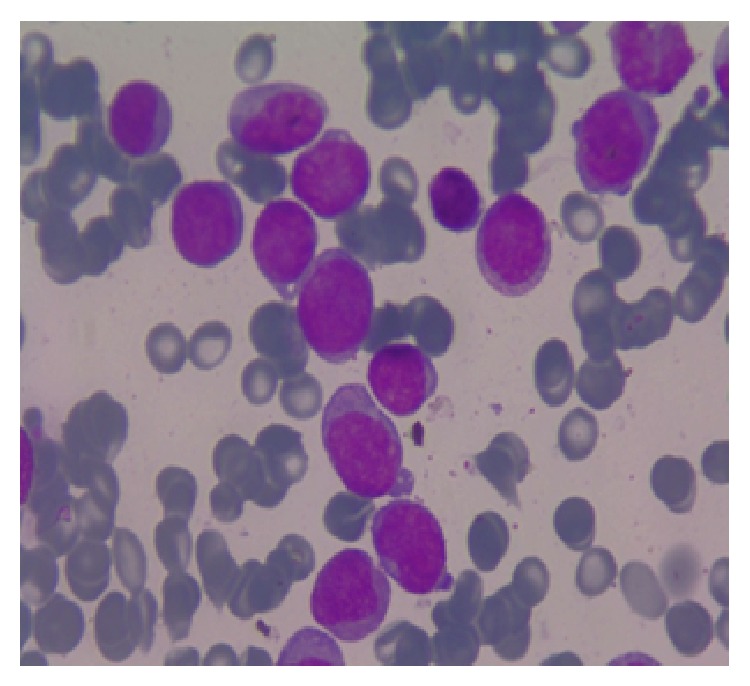
The view of myeloblasts on bone marrow aspiration material (×1000, Giemsa stain).

**Figure 3:**
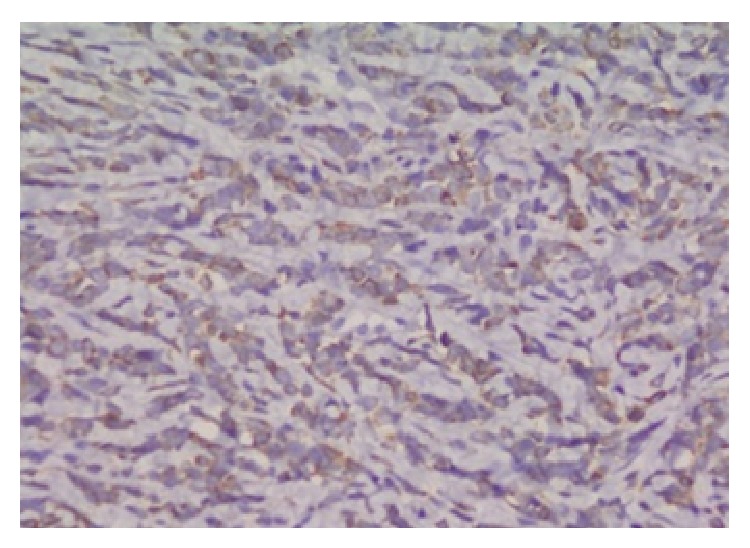
Immunohistochemistry showing MPO immunoreactivity in the cytoplasm of the neoplastic cells (×400, myeloperoxidase stain).

A 15-year-old girl was admitted to the emergency room with the complaint of abdominal pain for 3 days. On physical examination a solid mass was palpable on the left lower quadrant of the abdomen. She had neither organomegaly nor lymphadenopathy. On ultrasound examination, a uniform, well-demarcated mass with 70 × 58 mm size was seen on the left superolateral part of the uterus. On MRI examination, 92 × 70 × 80 mm sized, encapsulated, well-demarcated mass was observed at the same location (Figure 1). The right ovary was seen in both of the imagining techniques but the left one was not seen. Complete blood count revealed hyperleukocytosis with a leukocyte count of 100.6 × 10^9^/L, anemia (hemoglobin: 107 g/L), and mild thrombocytopenia (platelets: 125 × 10^9^/L). Lactate dehydrogenase level was 1144 U/L and other biochemical tests were at normal ranges. On peripheral blood smear examination 20% of cells were blasts. On bone marrow aspiration, 85% of cells were large myeloid blasts with fine chromatin and striking nucleoli (Figure 2). Eosinophilia or Auer rods were not observed. Since the rate of myeloid maturation was over 10%, findings of bone marrow aspiration were correlated with AML-M2 with maturation type. By flow cytometric analysis, 77% of mononuclear cells were positive for CD33, CD34, CD13, HLA-DR, CD117, and cytoplasmic MPO. CD14 was positive in 8% of the cells. Tru-cut biopsy was performed from the pelvic mass and there were atypical, large neoplastic cells with round, fine hyperchromatic nuclei spreading as single cell lines and in some areas they were showing cohesive aggregates. There was an extensive fibrotic stroma causing widespread squeezing artefact in neoplastic cells. CD34, CD117, MPO, and lysozyme were diffusely positive in neoplastic cells (Figure 3). These findings were consistent with myeloid sarcoma. By cytogenetic analysis inv(16) was positive and t(8; 21), t(15; 17), t(4; 11), and t(9; 22) were negative.

The induction chemotherapy of AML-BFM 2004 protocol was started. An MRI was performed just before the beginning of the third block and 60% of shrinkage was achieved. Apart from the third block of chemotherapy, high-dose methylprednisolone was given for 7 days [10]. Two weeks after this therapy, on ultrasound examination, the mass still persisted. Laparoscopy was performed and the mass was extracted, preserving the left ovarian tissue. It was verified that the mass was originating from the left ovary. Total necrosis was seen on pathological examination of the material. Our patient completed 5 blocks of AML-BFM 2004 protocol without any problem. On control bone marrow examinations there were no blasts and control inv(16) became negative. She is being followed up on her ninth month of the maintenance therapy.

## 3. Discussion

AML is a hematological malignancy characterized by proliferation of myeloid cell precursors with or without maturation. The initial symptoms of AML are related to anemia, neutropenia, and thrombocytopenia which develop due to bone marrow infiltration of leukemic blasts [1, 2]. Myeloid sarcoma is defined as the accumulation of immature myeloid cells or myeloblasts in the extramedullary area. It is seen in 1–5% of all AML cases. It is more probable to encounter myeloid sarcoma in childhood AML than adults [11]. In the report of Ohanian et al. myeloid sarcoma was seen in 9% of all age groups of AML cases and in 40% of childhood AML cases [12]. The signs and symptoms of myeloid sarcoma are related to pressure effect on the adjacent structures [4–6]. Our patient's initial symptom was related to myeloid sarcoma mass. Myeloid sarcomas are commonly located at bone, periost, soft tissues of head and neck region, skin, and orbita. Rarely they can be located at intestine, mediastinum, pleura, peritoneum, biliary tract, breast, uterus, and ovaries [4–6, 13]. There are reports of case series about orbitally located myeloid sarcomas in Turkish children [3]. If there are associated cytogenetic abnormalities like inv(16) or t(8; 21), myeloid sarcoma is more commonly encountered [4, 5, 14]. Our case had inv(16) and supports this information. At the time of diagnosis since the pelvic mass was very big, we could not figure out the origin of the mass. During laparoscopy it was clearly seen that the mass was originating from the left ovary.

AML with maturation (FAB AML-M2) comprises 10–15% of all AML cases. To fulfill morphological diagnostic criteria, in the bone marrow, blast percentage must be over 20%, mature myeloid cell percentage must be over 10%, and monocytic component must be less than 20%. Immunophenotypically expression of CD13, CD33, CD65, CD11b, CD15, CD34, CD38, and HLA-DR is present [15, 16].

By cytogenetic analysis in cases of AML-M2, generally t(8; 21) is present. Inv(16) and t(16; 16) which are abnormalities of the 16th chromosome are seen in 7-8% of AML cases [17–19]. Inv(16) generally accompanies AML-M4Eos, but as in our case there are reports of AML-M2 cases with inv(16) positivity. He et al. reported in their paper that, in 15 of inv(16) positive AML patients, 12 of them had AML-M4Eos and one of them had AML-M2 [20]. In the research of Chan et al. 5 of the 43 AML patients had inv(16) and 3 of them had AML-M2 [21].

The prognosis of AML patients with inv(16) is generally better than other AML subtypes. Because the response to chemotherapy is satisfactory, it is recommended that bone marrow transplantation should be reserved for relapsed cases [1, 2, 9]. In our case, after the third block of chemotherapy total remission was achieved and inv(16) became negative.

In conclusion, in children who present with a solid mass, the possibility of myeloid sarcoma should be kept in mind. Our case also supported the opinion that inv(16) is not restricted to AML-M4Eos subtype.
